# *catena*-Poly[[[bis­(methanol-κ*O*)lead(II)]-μ-*N*′-[1-(pyridin-2-yl-κ*N*)ethyl­idene]isonicotinohydrazidato-κ^3^ *N*′,*O*:*N* ^1^] perchlorate]

**DOI:** 10.1107/S1600536811046769

**Published:** 2011-11-09

**Authors:** Gholam Hossein Shahverdizadeh, Edward R. T. Tiekink, Babak Mirtamizdoust

**Affiliations:** a Department of Chemistry, Faculty of Science, Tabriz Branch, Islamic Azad University, PO Box 1655, Tabriz, Iran; b Department of Chemistry, University of Malaya, 50603 Kuala Lumpur, Malaysia; c Department of Inorganic Chemistry, Faculty of Chemistry, University of Tabriz, PO Box 5166616471, Tabriz, Iran

## Abstract

The Pb^II^ atom in the polymeric title compound, {[Pb(C_13_H_11_N_4_O)(CH_3_OH)_2_]ClO_4_}_*n*_, is coordinated by an *N*′-[1-(pyridin-2-yl-κ*N*)ethyl­idene]isonicotinohydrazidate ligand *via O*,*N*,*N*′-donors and simultaneously bridged by a neighbouring ligand *via* the isonicotinoyl N atom; two additional sites are occupied by methanol O atoms. The resultant supra­molecular chain is a zigzag along the *c* axis. The Pb^II^ atom is seven-coordinated within an N_3_O_3_ donor set and a lone pair of electrons, which defines a Ψ-pentagonal–bipyramidal coordination geometry with the pyridine N and lone pair in axial positions. The supra­molecular chains are linked into the two-dimensional array *via* inter­molecular Pb⋯N [3.020 (4) Å] inter­actions. Layers stack along the *a* axis, being connected by O—H⋯O hydrogen bonds formed between the coordinated methanol mol­ecules and perchlorate anions.

## Related literature

For the structures of metal complexes containing the *N*′-[1-(2-pyrid­yl)ethyl­idene]isonicotinohydrazide ligand, see: Maurya *et al.* (2002 ▶); Abboud *et al.* (2007 ▶); Zhang & Liu (2009 ▶); Hao *et al.* (2010 ▶); Shahverdizadeh *et al.* (2011 ▶). For specialized crystallization techniques, see: Harrowfield *et al.* (1996 ▶).
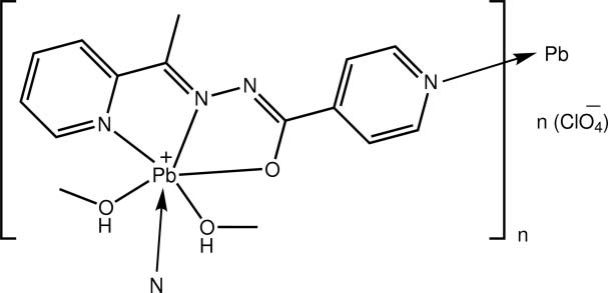


## Experimental

### 

#### Crystal data

[Pb(C_13_H_11_N_4_O)(CH_4_O)_2_]ClO_4_
*M* *_r_* = 609.98
Monoclinic, 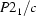

*a* = 11.2122 (14) Å
*b* = 13.4644 (17) Å
*c* = 14.1451 (18) Å
β = 111.906 (2)°
*V* = 1981.2 (4) Å^3^
*Z* = 4
Mo *K*α radiation
μ = 8.70 mm^−1^
*T* = 173 K
0.46 × 0.43 × 0.37 mm

#### Data collection

Bruker SMART CCD area-detector diffractometer
Absorption correction: multi-scan (*SADABS*; Sheldrick, 1996 ▶) *T*_min_ = 0.108, *T*_max_ = 0.141
10367 measured reflections
3492 independent reflections
2641 reflections with *I* > 2σ(*I*)
*R*_int_ = 0.027

#### Refinement

*R*[*F* ^2^ > 2σ(*F* ^2^)] = 0.024
*wR*(*F* ^2^) = 0.066
*S* = 1.05
3492 reflections
255 parameters
H-atom parameters constrained
Δρ_max_ = 0.97 e Å^−3^
Δρ_min_ = −0.98 e Å^−3^

### 

Data collection: *SMART* (Bruker, 2007 ▶); cell refinement: *SAINT* (Bruker, 2007 ▶); data reduction: *SAINT*; program(s) used to solve structure: *SHELXS97* (Sheldrick, 2008 ▶); program(s) used to refine structure: *SHELXL97* (Sheldrick, 2008 ▶); molecular graphics: *ORTEP-3* (Farrugia, 1997 ▶) and *DIAMOND* (Brandenburg, 2006 ▶); software used to prepare material for publication: *publCIF* (Westrip, 2010 ▶).

## Supplementary Material

Crystal structure: contains datablock(s) global, I. DOI: 10.1107/S1600536811046769/hg5133sup1.cif

Structure factors: contains datablock(s) I. DOI: 10.1107/S1600536811046769/hg5133Isup2.hkl

Additional supplementary materials:  crystallographic information; 3D view; checkCIF report

## Figures and Tables

**Table 1:** Selected bond lengths (Å)

Pb—O1	2.415 (3)
Pb—O2	2.733 (4)
Pb—O3	2.891 (4)
Pb—N1	2.669 (4)
Pb—N2	2.493 (4)
Pb—N4^i^	2.477 (4)

Symmetry code: (i) 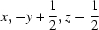.

**Table 2:** Hydrogen-bond geometry (Å, °)

*D*—H⋯*A*	*D*—H	H⋯*A*	*D*⋯*A*	*D*—H⋯*A*
O2—H2o⋯O4	0.84	2.16	2.909 (7)	149
O3—H3o⋯O5^ii^	0.84	2.11	2.930 (7)	166

Symmetry code: (ii) 
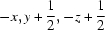
.

**Fig. 1.**
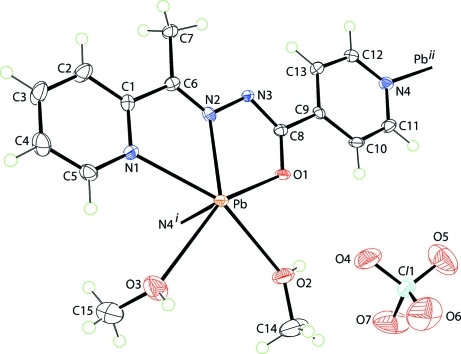
The asymmetric unit of (I) showing the atom-labelling scheme and displacement ellipsoids at the 50% probability level. The molecular structure has been expanded to indicate the µ2-bridging mode of the tetradentate ligand. Symmetry operations i: x, 0.5 - y, -1/2 + z; ii: x, 1/2 + y, 0.5 - z.

**Fig. 2.**
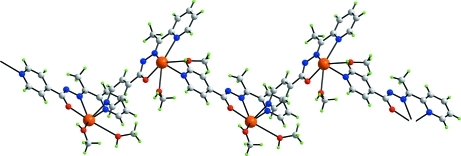
A view of the supramolecular zigzag chain along the c axis in (I).

**Fig. 3.**
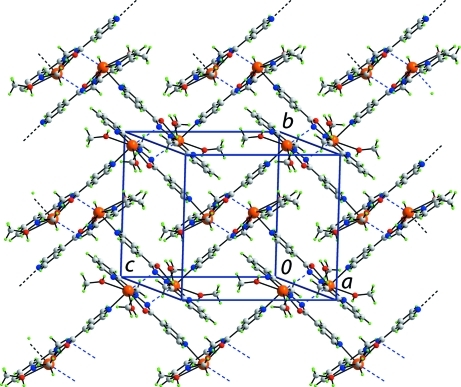
A view of the two-dimensional array in the bc plane in (I). The weaker Pb···N interactions (see text) are shown as blue dashed lines.

**Fig. 4.**
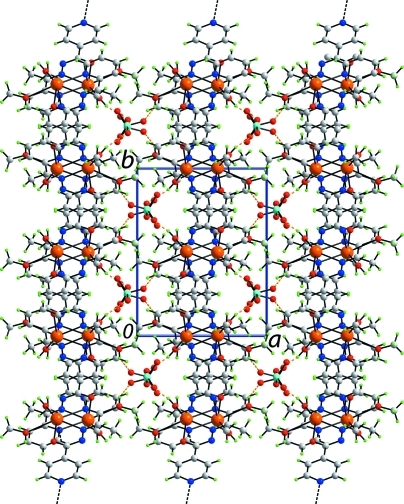
A view in projection down the c axis of the crystal packing in (I). The weaker Pb···N interactions (see text) are shown as blue dashed lines, and the O—H···O hydrogen bonds are shown as orange dashed lines.

## supplementary crystallographic information

### Comment

Structural studies of coordination complexes containing the *N*'-[1-(2-pyridyl)ethylidene]isonicotinohydrazide ligand are rare (Maurya *et al.*, 2002; Abboud *et al.*, 2007; Zhang & Liu, 2009; Hao *et al.*, 2010). In each of these, the ligand coordinates in a tridentate mode with the terminal 4-pyridyl-N atom being non-coordinating. In a recently reported Pb^II^ structure, all four donor atoms were involved in coordination leading to a zigzag chain (Shahverdizadeh *et al.*, 2011). A similar mode of coordination was found in the title lead(II) complex, (I), with complete details described herein.

The crystallographic asymmetric unit of (I), Fig. 1, comprises a Pb^II^ atom, an *N*'-1-(2-pyridyl)ethylidene]isonicotinohydrazide anion, a perchlorate anion, and two methanol molecules. The *N*'-[1-(2-pyridyl)ethylidene]isonicotinohydrazide ligand coordinates a lead atom in a tridentate mode, *via* the N1, N2 and O1 atoms, and simultaneously bridges a symmetry related lead atom *via* the 4-pyridyl-N4 atom., Table 1. Both methanol molecules are connected to the Pb^II^ atom. The resulting N_3_O_3_ donor set, along with a stereochemically active lone pair of electrons, define a pentagonal bipyramidal geometry with the pyridyl-N4 atom lone pair of electrons occupying axial positions. The µ_2_-bridging mode of the tetradentate *N*'-[1-(2-pyridyl)ethylidene]isonicotinohydrazide ligand leads to a zigzag chain (glide symmetry) along the *c* axis, Fig. 2.

In the crystal packing, centrosymmetrically related supramolecular chains are linked into a two-dimensional array by Pb···N interactions [Pb···N3^i^ = 3.020 (4) Å for *i*: 1 - *x*, 1 - *y*, 1 - *z*]. The layers thus formed in the *bc* plane are connected into a three-dimensional architecture *via* O—H···O hydrogen bonds involving the coordinated methanol molecules and the perchlorate anions, Fig. 4 and Table 2.

### Experimental

A solution of methyl 2-pyridyl ketone (15 mmol) in MeOH (25 ml) was added drop-wise to a solution of 4-pyridinecarboxylic acid hydrazide (15 mmol) in MeOH (15 ml). The mixture was refluxed for 6 h. The white precipitate was removed by filtration and recrystallized from MeOH solution. Then the compound (1 mmol) was placed in one arm of a branched tube (Harrowfield *et al.*, 1996) and a mixture of lead(II) acetate (1 mmol) and sodium perchlorate (1 mmol) in the other. Methanol was then added to fill both arms, the tube sealed and the ligand-containing arm immersed in a bath at 333 K, while the other was left at ambient temperature. After 2 weeks, crystals had deposited in the arm held at ambient temperature. They were filtered off, washed with acetone and ether, and air dried. Yield: 66%; *M*.pt.: 516 K.

### Refinement

The O– and C-bound H-atoms were placed in calculated positions [O—H = 0.84 Å; C—H = 0.95–0.98 Å, *U*_iso_(H) = 1.2–1.5*U*_eq_(parent atom)] and were included in the refinement in the riding model approximation. The atoms comprising the coordinated methanol molecules (and anion) exhibit greater thermal motion than the other atoms in the structure. No evidence for resolvable disorder was found, however.

### Crystal data

**Table:**

[Pb(C_13_H_11_N_4_O)(CH_4_O)_2_]ClO_4_	*F*(000) = 1168
*M**_r_* = 609.98	*D*_x_ = 2.045 Mg m^−^^3^
Monoclinic, *P*2_1_/*c*	Mo *K*α radiation, λ = 0.71073 Å
Hall symbol: -P 2ybc	Cell parameters from 4546 reflections
*a* = 11.2122 (14) Å	θ = 2.2–25.0°
*b* = 13.4644 (17) Å	µ = 8.70 mm^−^^1^
*c* = 14.1451 (18) Å	*T* = 173 K
β = 111.906 (2)°	Prism, yellow
*V* = 1981.2 (4) Å^3^	0.46 × 0.43 × 0.37 mm
*Z* = 4	

### Data collection

**Table:**

Bruker SMART CCD area-detector diffractometer	3492 independent reflections
Radiation source: fine-focus sealed tube	2641 reflections with *I* > 2σ(*I*)
graphite	*R*_int_ = 0.027
φ and ω scans	θ_max_ = 25.0°, θ_min_ = 2.0°
Absorption correction: multi-scan (*SADABS*; Sheldrick, 1996)	*h* = −13→8
*T*_min_ = 0.108, *T*_max_ = 0.141	*k* = −16→15
10367 measured reflections	*l* = −12→16

### Refinement

**Table:**

Refinement on *F*^2^	Primary atom site location: structure-invariant direct methods
Least-squares matrix: full	Secondary atom site location: difference Fourier map
*R*[*F*^2^ > 2σ(*F*^2^)] = 0.024	Hydrogen site location: inferred from neighbouring sites
*wR*(*F*^2^) = 0.066	H-atom parameters constrained
*S* = 1.05	*w* = 1/[σ^2^(*F*_o_^2^) + (0.0282*P*)^2^ + 6.1832*P*] where *P* = (*F*_o_^2^ + 2*F*_c_^2^)/3
3492 reflections	(Δ/σ)_max_ = 0.001
255 parameters	Δρ_max_ = 0.97 e Å^−^^3^
0 restraints	Δρ_min_ = −0.98 e Å^−^^3^

### Special details

**Table:**

Geometry. All s.u.'s (except the s.u. in the dihedral angle between two l.s. planes) are estimated using the full covariance matrix. The cell s.u.'s are taken into account individually in the estimation of s.u.'s in distances, angles and torsion angles; correlations between s.u.'s in cell parameters are only used when they are defined by crystal symmetry. An approximate (isotropic) treatment of cell s.u.'s is used for estimating s.u.'s involving l.s. planes.
Refinement. Refinement of *F*^2^ against ALL reflections. The weighted *R*-factor *wR* and goodness of fit *S* are based on *F*^2^, conventional *R*-factors *R* are based on *F*, with *F* set to zero for negative *F*^2^. The threshold expression of *F*^2^ > 2σ(*F*^2^) is used only for calculating *R*-factors(gt) *etc*. and is not relevant to the choice of reflections for refinement. *R*-factors based on *F*^2^ are statistically about twice as large as those based on *F*, and *R*- factors based on ALL data will be even larger.

### Fractional atomic coordinates and isotropic or equivalent isotropic displacement parameters (Å^2^)

**Table:**

	*x*	*y*	*z*	*U*_iso_*/*U*_eq_	
Pb	0.379666 (16)	0.502370 (14)	0.303341 (13)	0.01308 (8)	
Cl1	−0.07646 (14)	0.25586 (11)	0.34029 (12)	0.0297 (3)	
O1	0.3551 (3)	0.3685 (3)	0.4082 (3)	0.0160 (8)	
O2	0.1391 (4)	0.4209 (3)	0.2361 (3)	0.0304 (10)	
H2O	0.1367	0.3678	0.2666	0.037*	
O3	0.2933 (5)	0.5804 (4)	0.0983 (3)	0.0449 (12)	
H3O	0.2399	0.6262	0.0916	0.054*	
O4	0.0480 (5)	0.2805 (5)	0.3505 (4)	0.080 (2)	
O5	−0.0732 (6)	0.2110 (5)	0.4315 (4)	0.081 (2)	
O6	−0.1531 (6)	0.3419 (5)	0.3194 (5)	0.088 (2)	
O7	−0.1306 (6)	0.1904 (5)	0.2568 (4)	0.076 (2)	
N1	0.6011 (4)	0.5630 (3)	0.2931 (3)	0.0171 (10)	
N2	0.5835 (4)	0.4394 (3)	0.4342 (3)	0.0138 (9)	
N3	0.5741 (4)	0.3751 (3)	0.5074 (3)	0.0138 (9)	
N4	0.4129 (4)	0.1305 (3)	0.6945 (3)	0.0168 (10)	
C1	0.7110 (5)	0.5362 (4)	0.3701 (4)	0.0136 (11)	
C2	0.8302 (5)	0.5696 (4)	0.3747 (5)	0.0255 (13)	
H2	0.9062	0.5498	0.4293	0.031*	
C3	0.8377 (6)	0.6317 (5)	0.2996 (5)	0.0313 (15)	
H3	0.9188	0.6541	0.3011	0.038*	
C4	0.7259 (6)	0.6607 (4)	0.2223 (5)	0.0285 (14)	
H4	0.7282	0.7047	0.1705	0.034*	
C5	0.6108 (5)	0.6249 (4)	0.2216 (4)	0.0205 (12)	
H5	0.5340	0.6448	0.1679	0.025*	
C6	0.6980 (5)	0.4684 (4)	0.4476 (4)	0.0147 (11)	
C7	0.8175 (5)	0.4367 (4)	0.5349 (4)	0.0195 (12)	
H7A	0.7934	0.3963	0.5826	0.029*	
H7B	0.8646	0.4956	0.5701	0.029*	
H7C	0.8722	0.3975	0.5087	0.029*	
C8	0.4546 (5)	0.3432 (4)	0.4834 (4)	0.0125 (11)	
C9	0.4411 (5)	0.2683 (4)	0.5577 (4)	0.0120 (10)	
C10	0.3203 (5)	0.2332 (4)	0.5473 (4)	0.0163 (11)	
H10	0.2457	0.2561	0.4932	0.020*	
C11	0.3101 (5)	0.1652 (4)	0.6160 (4)	0.0182 (12)	
H11	0.2271	0.1414	0.6080	0.022*	
C12	0.5287 (5)	0.1646 (4)	0.7041 (4)	0.0174 (12)	
H12	0.6019	0.1410	0.7590	0.021*	
C13	0.5467 (5)	0.2326 (4)	0.6377 (4)	0.0148 (11)	
H13	0.6307	0.2546	0.6468	0.018*	
C15	0.0171 (6)	0.4465 (6)	0.1622 (6)	0.0463 (19)	
H15A	−0.0462	0.3962	0.1617	0.069*	
H15B	0.0228	0.4497	0.0948	0.069*	
H15C	−0.0094	0.5114	0.1791	0.069*	
C16	0.3117 (8)	0.5665 (8)	0.0072 (6)	0.065 (3)	
H16A	0.3963	0.5365	0.0213	0.098*	
H16B	0.3071	0.6307	−0.0265	0.098*	
H16C	0.2447	0.5224	−0.0373	0.098*	

### Atomic displacement parameters (Å^2^)

**Table:**

	*U*^11^	*U*^22^	*U*^33^	*U*^12^	*U*^13^	*U*^23^
Pb	0.01177 (12)	0.01436 (11)	0.01108 (11)	0.00120 (9)	0.00192 (8)	−0.00026 (9)
Cl1	0.0206 (7)	0.0349 (8)	0.0335 (8)	−0.0006 (7)	0.0101 (6)	−0.0074 (7)
O1	0.013 (2)	0.020 (2)	0.0106 (18)	−0.0043 (15)	−0.0003 (15)	0.0014 (15)
O2	0.018 (2)	0.037 (2)	0.028 (2)	−0.0036 (18)	0.0004 (18)	0.002 (2)
O3	0.046 (3)	0.057 (3)	0.035 (3)	0.024 (2)	0.018 (2)	0.015 (2)
O4	0.028 (3)	0.143 (6)	0.057 (4)	−0.022 (3)	0.003 (3)	0.039 (4)
O5	0.099 (5)	0.097 (5)	0.048 (4)	−0.042 (4)	0.031 (3)	0.000 (3)
O6	0.078 (5)	0.084 (5)	0.098 (5)	0.040 (4)	0.027 (4)	−0.015 (4)
O7	0.078 (4)	0.075 (4)	0.050 (4)	0.013 (3)	−0.006 (3)	−0.032 (3)
N1	0.013 (2)	0.021 (2)	0.017 (2)	−0.0003 (19)	0.0044 (19)	0.0029 (19)
N2	0.018 (2)	0.011 (2)	0.013 (2)	0.0007 (18)	0.0071 (18)	0.0020 (17)
N3	0.010 (2)	0.019 (2)	0.011 (2)	−0.0015 (18)	0.0024 (18)	0.0029 (18)
N4	0.017 (2)	0.017 (2)	0.015 (2)	0.0003 (19)	0.0042 (19)	0.0030 (18)
C1	0.018 (3)	0.009 (2)	0.017 (3)	0.000 (2)	0.010 (2)	−0.003 (2)
C2	0.014 (3)	0.027 (3)	0.033 (3)	0.004 (2)	0.006 (3)	0.010 (3)
C3	0.021 (3)	0.033 (4)	0.043 (4)	0.000 (3)	0.015 (3)	0.017 (3)
C4	0.036 (4)	0.023 (3)	0.030 (4)	0.000 (3)	0.016 (3)	0.010 (3)
C5	0.023 (3)	0.020 (3)	0.017 (3)	0.000 (2)	0.005 (2)	0.005 (2)
C6	0.013 (3)	0.013 (2)	0.017 (3)	0.002 (2)	0.004 (2)	−0.001 (2)
C7	0.012 (3)	0.026 (3)	0.019 (3)	0.003 (2)	0.003 (2)	0.006 (2)
C8	0.013 (3)	0.012 (3)	0.012 (3)	0.000 (2)	0.003 (2)	−0.003 (2)
C9	0.015 (3)	0.012 (2)	0.010 (2)	−0.002 (2)	0.005 (2)	−0.003 (2)
C10	0.013 (3)	0.020 (3)	0.011 (3)	−0.002 (2)	0.000 (2)	0.000 (2)
C11	0.013 (3)	0.020 (3)	0.020 (3)	−0.005 (2)	0.004 (2)	0.000 (2)
C12	0.016 (3)	0.021 (3)	0.014 (3)	0.000 (2)	0.003 (2)	0.000 (2)
C13	0.013 (3)	0.014 (3)	0.017 (3)	0.000 (2)	0.005 (2)	0.001 (2)
C15	0.024 (4)	0.056 (5)	0.044 (4)	0.006 (3)	−0.004 (3)	−0.005 (4)
C16	0.043 (5)	0.116 (8)	0.031 (4)	0.004 (5)	0.007 (4)	0.005 (5)

### Geometric parameters (Å, °)

**Table:**

Pb—O1	2.415 (3)	C2—H2	0.9500
Pb—O2	2.733 (4)	C3—C4	1.378 (8)
Pb—O3	2.891 (4)	C3—H3	0.9500
Pb—N1	2.669 (4)	C4—C5	1.374 (8)
Pb—N2	2.493 (4)	C4—H4	0.9500
Pb—N4^i^	2.477 (4)	C5—H5	0.9500
Cl1—O4	1.389 (5)	C6—C7	1.505 (7)
Cl1—O6	1.406 (6)	C7—H7A	0.9800
Cl1—O5	1.412 (6)	C7—H7B	0.9800
Cl1—O7	1.417 (5)	C7—H7C	0.9800
O1—C8	1.268 (6)	C8—C9	1.505 (7)
O2—C15	1.420 (7)	C9—C13	1.384 (7)
O2—H2O	0.8400	C9—C10	1.389 (7)
O3—C16	1.392 (9)	C10—C11	1.371 (7)
O3—H3O	0.8400	C10—H10	0.9500
N1—C5	1.345 (7)	C11—H11	0.9500
N1—C1	1.354 (7)	C12—C13	1.379 (7)
N2—C6	1.286 (7)	C12—H12	0.9500
N2—N3	1.384 (6)	C13—H13	0.9500
N3—C8	1.325 (7)	C15—H15A	0.9800
N4—C12	1.335 (7)	C15—H15B	0.9800
N4—C11	1.351 (7)	C15—H15C	0.9800
N4—Pb^ii^	2.477 (4)	C16—H16A	0.9800
C1—C2	1.389 (7)	C16—H16B	0.9800
C1—C6	1.475 (8)	C16—H16C	0.9800
C2—C3	1.379 (8)		
			
O1—Pb—N4^i^	85.45 (13)	C2—C3—H3	120.5
O1—Pb—N2	64.74 (12)	C5—C4—C3	118.7 (5)
N4^i^—Pb—N2	84.66 (14)	C5—C4—H4	120.7
O1—Pb—N1	125.71 (12)	C3—C4—H4	120.7
N4^i^—Pb—N1	80.14 (14)	N1—C5—C4	123.4 (5)
N2—Pb—N1	61.99 (13)	N1—C5—H5	118.3
O1—Pb—O2	65.79 (12)	C4—C5—H5	118.3
N4^i^—Pb—O2	81.46 (14)	N2—C6—C1	116.6 (5)
N2—Pb—O2	129.38 (13)	N2—C6—C7	124.6 (5)
N1—Pb—O2	157.14 (13)	C1—C6—C7	118.8 (5)
O1—Pb—O3	144.62 (13)	C6—C7—H7A	109.5
N4^i^—Pb—O3	73.20 (14)	C6—C7—H7B	109.5
N2—Pb—O3	137.72 (13)	H7A—C7—H7B	109.5
N1—Pb—O3	78.73 (13)	C6—C7—H7C	109.5
O2—Pb—O3	83.06 (14)	H7A—C7—H7C	109.5
O4—Cl1—O6	109.6 (5)	H7B—C7—H7C	109.5
O4—Cl1—O5	108.9 (4)	O1—C8—N3	128.2 (5)
O6—Cl1—O5	110.5 (4)	O1—C8—C9	118.6 (5)
O4—Cl1—O7	109.7 (4)	N3—C8—C9	113.2 (4)
O6—Cl1—O7	107.4 (4)	C13—C9—C10	118.1 (5)
O5—Cl1—O7	110.6 (4)	C13—C9—C8	121.7 (5)
C8—O1—Pb	116.7 (3)	C10—C9—C8	120.2 (5)
C15—O2—Pb	135.5 (4)	C11—C10—C9	119.2 (5)
C15—O2—H2O	112.3	C11—C10—H10	120.4
Pb—O2—H2O	112.3	C9—C10—H10	120.4
C16—O3—Pb	140.3 (5)	N4—C11—C10	122.8 (5)
C16—O3—H3O	109.8	N4—C11—H11	118.6
Pb—O3—H3O	109.8	C10—C11—H11	118.6
C5—N1—C1	117.8 (5)	N4—C12—C13	122.9 (5)
C5—N1—Pb	124.6 (3)	N4—C12—H12	118.6
C1—N1—Pb	117.4 (3)	C13—C12—H12	118.6
C6—N2—N3	115.5 (4)	C12—C13—C9	119.3 (5)
C6—N2—Pb	126.5 (3)	C12—C13—H13	120.3
N3—N2—Pb	117.6 (3)	C9—C13—H13	120.3
C8—N3—N2	111.3 (4)	O2—C15—H15A	109.5
C12—N4—C11	117.6 (4)	O2—C15—H15B	109.5
C12—N4—Pb^ii^	123.4 (3)	H15A—C15—H15B	109.5
C11—N4—Pb^ii^	118.7 (3)	O2—C15—H15C	109.5
N1—C1—C2	121.5 (5)	H15A—C15—H15C	109.5
N1—C1—C6	116.8 (5)	H15B—C15—H15C	109.5
C2—C1—C6	121.7 (5)	O3—C16—H16A	109.5
C3—C2—C1	119.6 (5)	O3—C16—H16B	109.5
C3—C2—H2	120.2	H16A—C16—H16B	109.5
C1—C2—H2	120.2	O3—C16—H16C	109.5
C4—C3—C2	119.0 (6)	H16A—C16—H16C	109.5
C4—C3—H3	120.5	H16B—C16—H16C	109.5
			
N4^i^—Pb—O1—C8	94.7 (4)	C5—N1—C1—C2	−1.2 (8)
N2—Pb—O1—C8	8.5 (3)	Pb—N1—C1—C2	−175.8 (4)
N1—Pb—O1—C8	20.3 (4)	C5—N1—C1—C6	−180.0 (5)
O2—Pb—O1—C8	177.4 (4)	Pb—N1—C1—C6	5.4 (6)
O3—Pb—O1—C8	147.0 (3)	N1—C1—C2—C3	0.2 (9)
O1—Pb—O2—C15	168.6 (6)	C6—C1—C2—C3	178.9 (5)
N4^i^—Pb—O2—C15	−102.5 (6)	C1—C2—C3—C4	1.2 (9)
N2—Pb—O2—C15	−178.4 (5)	C2—C3—C4—C5	−1.4 (10)
N1—Pb—O2—C15	−65.8 (7)	C1—N1—C5—C4	0.9 (8)
O3—Pb—O2—C15	−28.6 (6)	Pb—N1—C5—C4	175.1 (4)
O1—Pb—O3—C16	−79.2 (8)	C3—C4—C5—N1	0.4 (9)
N4^i^—Pb—O3—C16	−23.8 (8)	N3—N2—C6—C1	179.5 (4)
N2—Pb—O3—C16	37.7 (9)	Pb—N2—C6—C1	−8.2 (6)
N1—Pb—O3—C16	59.1 (8)	N3—N2—C6—C7	0.4 (7)
O2—Pb—O3—C16	−107.0 (8)	Pb—N2—C6—C7	172.8 (4)
O1—Pb—N1—C5	167.4 (4)	N1—C1—C6—N2	1.1 (7)
N4^i^—Pb—N1—C5	90.3 (4)	C2—C1—C6—N2	−177.7 (5)
N2—Pb—N1—C5	179.5 (5)	N1—C1—C6—C7	−179.8 (5)
O2—Pb—N1—C5	53.5 (6)	C2—C1—C6—C7	1.4 (8)
O3—Pb—N1—C5	15.6 (4)	Pb—O1—C8—N3	−6.4 (7)
O1—Pb—N1—C1	−18.5 (4)	Pb—O1—C8—C9	173.2 (3)
N4^i^—Pb—N1—C1	−95.5 (4)	N2—N3—C8—O1	−3.9 (7)
N2—Pb—N1—C1	−6.3 (3)	N2—N3—C8—C9	176.6 (4)
O2—Pb—N1—C1	−132.3 (4)	O1—C8—C9—C13	175.8 (5)
O3—Pb—N1—C1	−170.2 (4)	N3—C8—C9—C13	−4.6 (7)
O1—Pb—N2—C6	177.0 (5)	O1—C8—C9—C10	−4.4 (7)
N4^i^—Pb—N2—C6	89.5 (4)	N3—C8—C9—C10	175.2 (5)
N1—Pb—N2—C6	7.8 (4)	C13—C9—C10—C11	0.0 (7)
O2—Pb—N2—C6	163.8 (4)	C8—C9—C10—C11	−179.8 (5)
O3—Pb—N2—C6	31.8 (5)	C12—N4—C11—C10	−0.3 (8)
O1—Pb—N2—N3	−10.8 (3)	Pb^ii^—N4—C11—C10	−175.1 (4)
N4^i^—Pb—N2—N3	−98.3 (3)	C9—C10—C11—N4	0.4 (8)
N1—Pb—N2—N3	−180.0 (4)	C11—N4—C12—C13	−0.1 (8)
O2—Pb—N2—N3	−24.0 (4)	Pb^ii^—N4—C12—C13	174.4 (4)
O3—Pb—N2—N3	−156.0 (3)	N4—C12—C13—C9	0.4 (8)
C6—N2—N3—C8	−175.1 (4)	C10—C9—C13—C12	−0.4 (7)
Pb—N2—N3—C8	11.8 (5)	C8—C9—C13—C12	179.4 (5)

Symmetry codes: (i) *x*, −*y*+1/2, *z*−1/2; (ii) *x*, −*y*+1/2, *z*+1/2.

### Hydrogen-bond geometry (Å, °)

**Table:**

*D*—H···*A*	*D*—H	H···*A*	*D*···*A*	*D*—H···*A*
O2—H2o···O4	0.84	2.16	2.909 (7)	149
O3—H3o···O5^iii^	0.84	2.11	2.930 (7)	166

Symmetry codes: (iii) −*x*, *y*+1/2, −*z*+1/2.
